# Multilevel analysis of determinants of cattle deaths in Ethiopia

**DOI:** 10.1371/journal.pone.0306434

**Published:** 2025-03-31

**Authors:** Yitagesu Kifelew Gizaw, A. R. Muralidharan, Zenebe Abebe Gebreegziabher, Biniyam Shumye Tefera, Alazar Gebeyehu

**Affiliations:** 1 Department of Statistics, College of Natural and Computational Science, Oda Bultum University, Chiro, Ethropia; 2 Department of Community Medicine, BGS Medical College and Hospital (BGS—MCH), BGS Vijnatham Campus, Bengaluru, Karnataka, India; 3 Department of Epidemiology and Biostatistics, School of Public Health, Asrat Woldeyes Health Science Campus, Debre Berhan University, Debre Berhan, Ethiopia; University of Uyo, NIGERIA

## Abstract

In Ethiopia, agriculture is a fundamental element of both the economy and the social fabric of the community. The sector employs 80-85 percent of the population and contributes 47% to the total GDP. Livestock contributes to people’s livelihoods through numerous channels: income, food, employment, transport, draft-over, manure, savings and insurance, social status, etc. Ethiopia is believed to have the largest livestock population in Africa. Despite this productive and reproductive performance is accompanied by poor health care, high disease incidence, poor management conditions, and unpredictable climactic conditions causing a significant cause of cattle death. The dependent variable is the count “number of occurrences of cattle death” that occurs randomly over time. A multilevel analysis was carried out with the anticipation that there would be variations in the number of cattle deaths per household throughout the region. Before analyzing the data with a multilevel method, check the variability using intra-class correlation (ICC), revealing that 14.6% of the variance in cattle deaths is attributable to the grouping level (Region) indicating the heterogeneity of cattle deaths between Regions. The multilevel ZINB regression model was identified as the best fit for analyzing cattle deaths per household. Factors such as types of agriculture, feeding areas, treatment methods, vaccination status, household land size, age of the household head, household size, and education level were found to significantly impact cattle mortality in the positive count portion of the random-intercept ZINB regression model. The Ministry of Agriculture should effectively raise awareness among agricultural producers regarding cattle vaccination and enhance the veterinary services available in the country. It is also advisable to promote a mixed farming approach rather than solely focusing on livestock farming to reduce cattle deaths. Farmers should consider reducing their household size and place greater emphasis on the welfare of their cattle.

## Introduction

Globally, Livestock is important for rural livelihoods and the economies of developing nations, serving as sources of income and employment for producers and other individuals involved in intricate value chains [1]. Livestock is a major contributor to food and nutritional security and serves as an important source of livelihood for nearly one billion poor people in developing countries [2]. Numerous livestock development policies and programs for sub-Saharan African economies tend to contribute to livestock production and productivity for poverty reduction and food security among rural households [3]. The agricultural sector has a major role in the Ethiopian economy and produces much of the nation’s food, approximately 80% to 85% of the workforce is employed in this industry, which also accounts for 47% of the GDP [4].

In Ethiopia, livestock represents a major national resource and an integral part of the agricultural production system, and livestock development is considered to be fundamental to the sustainable growth and transformation of Ethiopia [5]. Ethiopia has paid considerable attention to livestock productivity through breeding and health interventions to increase the contribution of livestock to economic growth [6]. However, in the last decade, the majority of the growth in the cattle subsector in Ethiopia has been due to increased numbers of animals or farmers rather than productivity improvements. To improve the sustainability of livestock growth in Ethiopia, a shift to improved productivity is required [7]. Among livestock species, cattle contribute to farmers’ livelihoods. They serve as a source of draught power for the rural farming population, supply farm families with milk, meat, and manure, and provide cash income, playing a significant role in society’s social and cultural values. Cattle provide almost all the draft power necessary for agricultural activities among smallholder farmers in Ethiopia [8].

Notwithstanding this, productivity and reproductive performance are accompanied by low levels of well-being, high rates of sickness, inadequate management practices, high disease incidence, and erratic weather patterns, all of which contribute significantly to the death of cattle [9]. If a household loses cattle, the result may be a loss of assets for a considerable period [10]. Livestock death is considered one of the main factors contributing to poverty [1].

Ethiopia has the largest livestock population in Africa with 65,354,090 heads of cattle, out of the total population the female cattle are 55.9% and the remaining 44.10% are male cattle [11]. However, the productive and reproductive performance is accompanied by low, poor health care, high disease incidence, poor management conditions, and unpredictable climatic conditions causing a significant cause for cattle death and still causing a negative influence on national economic activities [12,13].

Mortality among cattle is a serious problem that leads to financial losses. Based on data from the Ethiopian Central Statistics Agency, 3,109,310 cattle died between November 11, 2019, and November 10, 2020 [14]. High mortality leads to not only a loss of income but also a loss of replacement stock and genetic material, making it difficult for farmers to replace their losses or expand their herds [7,15]. Efficient production and limited losses are important for livestock producers to realize benefits from their livestock resources. To minimize losses, the causes of animal morbidity and mortality and the associated risk factors need to be identified, and appropriate control measures are implemented [15].

Researchers studied the determinate of cattle deaths using some set of variables and analysis using descriptive statistics, and chi-square (χ2) correlation analysis [12]. And statistical methods such as logistics regression models [15–18]. Many researchers prefer to categorize count variables as a binary and do analysis. In the binary probit model, commonly employed for examining cattle datasets, count variables are converted into binary variables [15]. However, the binary probit model undercounts the total mortality since multiple mortalities are collapsed into a single unit to fulfill the requirements of the binary probit model. Besides, the binary probit model cannot provide sufficient information for studying the pattern of multiple cattle deaths. It merely predicts the death or life of cattle rather than the number of cattle deaths. But in the study of numbers of cattle deaths, count regression models are more appropriate than other models and most of those researches are done on small-scale survey data come from only certain regions of the country. Researchers who studied the determinants of cattle deaths generally have a limited focus on considering appropriate models based on a regional variation of cattle death. Based on an agricultural sample survey of cattle mortality in Ethiopia, we employed multilevel count regression models to analyze the number of cattle deaths in this study.

The primary goal of this study was to use a multilevel logistic regression model to discover the variables that influence cattle death in Ethiopia.

- To investigate the risk factors of cattle deaths per household in Ethiopia.
- To identify factors that may explain the variation of cattle death between regions.
- To choose the best count regression model to use with the cattle death dataset analysis.

### Literature review

#### Livestock production systems.

**Fig 1:**
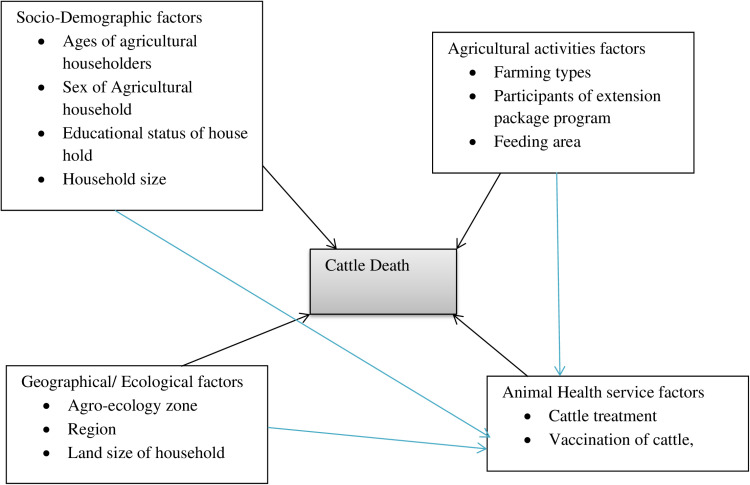
Conceptual framework on multilevel analysis of determinants of cattle deaths in Ethiopia.

Livestock farming occurs worldwide, offering a diverse range of products and services, utilizing different animal species and resources, and operating under a broad array of agro-ecological and socioeconomic scenarios [2]. As shown in Fig 1, The diversity of Ethiopia’s topography, climate, and cultural conditions make it difficult to generalize about cattle production systems in the country. Numerous authors used different criteria to classify livestock production systems in Ethiopia. In the highland area, a mixed farming system encompasses nearly 40% of the country’s land area. It features a mixed farming system where crop cultivation and livestock production are undertaken side by side and complement each other [8].

Providing adequate animal health services to smallholder farmers in developing countries has remained a challenge, despite various reform efforts during the past decades [19]. The focus of the past reforms was on market failures to decide what the public sector, the private sector, and the “third sector” (the community-based sector) should do about providing animal health services. However, such frameworks have paid limited attention to the governance challenges inherent in animal health services. The strong public sector involvement, especially in building and strengthening a synergistic relation-based referral arrangement between paraprofessionals and veterinarians is imperative in improving animal health service delivery in developing countries [20].

Livestock agriculture takes on many forms globally, providing products and services, employing different animal species and resources, and functioning within agroecological and socioeconomic contexts [21]. The potential for increased cattle production is proportionally lowered by various cattle management problems, major endemic diseases, poor feeding, and high stocking rates on grazing lands [8]. Mortality and Morbidity were higher in Dalocha and Sululta districts in mixed crop-livestock and peri-urban production systems, respectively [18]. The attendance of a farmer at a training course appeared to be protective against the mortality of his or her animals. The effect of training on mortality may have been due to early recognition and treatment of clinical cases [22]. Lack of access to adequate fodder and pasture has been reported as a management constraint in relatively small areas of the Kingdom of Bhutan [23]. The utilization of vaccination to protect their livestock herds against priority infectious diseases, while increased resilience to adversity, as well as the experience of diseases in herds, are positively associated with vaccination use [24]. However, vaccine supply and utilization rates by farmers in many sub-Saharan African countries including Ghana remain very low [25].

#### Empirical studies.

Over the years, several studies have been conducted on calf morbidity and mortality and associated risk factors in Ethiopia. According to a systematic review and meta-analysis of calf morbidity and mortality studies in Ethiopia, calf mortality ranges between 0.9% and 37%, with a pooled prevalence of 14.79% [26]. This study was conducted to identify factors associated with cattle death in dairy farming in Sweden using a negative binomial regression model. The study revealed that herd level, cattle size, calving interval, season, education level, and milk yield were associated with the death of dairy cattle [27].

The basic idea of multilevel analysis is that datasets with a nesting structure that includes unexplained variability at each nesting level are usually not adequately represented by single-level regression. The reason is that the unexplained variability in single-level Regression analysis is only the variance of the residual term. Variability in multilevel data, however, has a more complicated structure related to the fact that several populations are involved in modeling such data; one population for each Explain variability in a multilevel structure can be achieved by explaining variability between level-1 units but also explaining variability between higher-level units [28]. The following is a necessary notation to specify the multilevel regression model in this study. Let Yij represent the measure of the response variable Y for the individual I in the group. Index j = 1,2,……., N denotes higher level units(groups) and indexi = 1,2,…., N_j_ denotes the measures of the individual at group j. let X_p,ij,_ p = 1,2,…..,p, the p covariates included in the analysis, as measured at household *i* belonging to group j, $\beta_{0j}$ is assumed to vary randomly and given by the sum of an average intercept $\beta_{0}$ and group dependent deviations $U_{0j}$ (assumed mutually independent and normally distributed with mean zero and variance $\delta_{0}^{2}$), that is, $\beta_{0}+\sum_{p=1}^{p}\beta_{p}X_{pij}$, P = 1,2,….P, denotes slope coefficients and $\varepsilon_{ij}$ is a residualterm, assumed to have mean zero and variance $\delta_{\epsilon }^{2}$. Then we have the following types of models;

**Intercept-only model:** This is the simple case of a hierarchical linear model in which there are no explanatory variables at all. This model only contains random groups and variation within groups. It can be expressed as a model where the dependent variable is the sum general mean, $\beta_{0}$ a random effect at the group level $U_{oj}$ and a random effect at individual level $\varepsilon_{ij}$ that is,


$$y_{ij}=\beta_{0j}+\varepsilon_{ij}=\beta_{0}+U_{oj}+\varepsilon_{ij}$$ (1)

For intercept-only model $E\left(y_{ij}\right)=\beta_{0j}$ and $Var\left(U_{oj}\right)+var\left(\varepsilon_{ij}\right)=\delta_{0}^{2}+\delta_{\varepsilon }^{2}$ $Cov\left(y_{ij,y_{i'j}}\right)=var\left(U_{oj}\right)=\delta_{0}^{2}$ (The covariance b/n two individual (i≠i') in the same group

**Random intercept model:** This is a model in which all lower-level explanatory variables are fixed. This means that the corresponding variance components of the slope are fixed at zero. It is used to assess the contribution of each explanatory variable.


$$y_{ij}=\beta_{0}+\sum_{p=1}^{p}\beta_{p}X_{pij}$$ (2)

**Random intercept and coefficient model:** This is used to assess whether the slope of any of the explanatory variables has a significant variance component between the groups.


$$y_{ij}=\beta_{0}+\sum_{p=1}^{p}\beta_{p}X_{pij}+U_{0j}+\sum_{p=1}^{p}U_{pj}X_{pij}+\varepsilon_{ij}$$ (3)

The first part, $\beta_{0}+\sum_{p=1}^{p}\beta_{p}X_{pij}+U_{0j}$ is called a fixed part of the model and the second part, $\sum_{p=1}^{p}U_{pj}X_{pij}+\varepsilon_{ij}$ is called the random part. The term $\sum_{p=1}^{p}U_{pj}X_{pij}$ can be regarded as a random interaction between group and explanatory variables. This model implies that the groups are characterized by two rando effects; their intercept and their slopes. It assumes that for different groups, the pairs of random effects; ($U_{0j},U_{pj}$, P = 1,2…,p) are independent and identically distributed that they are independent of the level-1 residuals, $\varepsilon_{ij}$ and that $\varepsilon_{ij}$ are independent and identically distributed.

**Test of heterogeneity:** Intra-class correlation coefficient (ICC) represents the proportion of the total variance that is used to test heterogeneity in groups and it provides an assessment of whether or not significant between-groups variation exists. Then the intra-class correlation coefficient (ICC) at the region level is given by


$$CC=p=\frac{{\text{σ}}_{u}^{2}}{\sigma_{u}^{2}+\sigma_{e}^{2}}$$ (4)

where ${\text{σ}}_{u}^{2}$ is the between-group variance which can be estimated by within-group variance [29]

The Zero-inflated negative binomial (ZINB) regression model can handle Over-dispersed data with excess zeros. However, in hierarchical study design/nested data, both over-dispersion and lack of independence may occur [30]. Therefore, in these situations multilevel zero-inflated negative binomial random variable Y as


$$P\left(Y_{ij}=y_{ij}\right)= \{\begin{array}{cc} \omega_{ij}+\left(1-\omega_{ij}\right){\left(1+\theta_{\mu_{Ij}}\right)}^{\frac{-1}{\theta }}, & y_{ij}=0\\ {\left(1-\omega_{ij}\right)}^{\frac{\text{Γ}\left(y_{Ij+}\frac{1}{\theta }\right)+{\left(1+\frac{1}{\theta_{\mu_{I}j}}\right)}^{-y_{ij}}{\left(1+\theta_{\mu_{Ij}}\right)}^{\frac{-1}{\theta }}}{yij!\text{Γ}\left(y_{Ij+}\frac{1}{\theta }\right)},} & y_{ij}\geq 0 \end{array},\;0\leq \omega_{ij}\leq 1$$ (5)

The log and logit link functions are respectively given by


$$log\left(\mu_{ij}\right)=\log\left(n_{ij}\right)+x_{hij}^{,}\beta$$ (6)

$$logit\left(\omega_{ij}\right)=log\left(\frac{\omega_{ij}}{1-\omega_{ij}}\right)=\log\left(n_{ij}\right)+z_{hij}^{,}\gamma$$

## Materials and methods

### Source of data

The source of data for this study is secondary data from the Ethiopia Central Statistics Agency, an agricultural sample survey conducted from November 12, 2019, to November 11, 2020 [14].

### Sample size determination

We used all survey success data for 30576 agricultural householders who had cattle during the study period.

### Variables in the study

#### Response variable.

The response variable of this study was the number of cattle deaths in Ethiopia. For the current analysis, the response variable count response.

#### Explanatory variables.

The factors that could influence cattle mortality include the age of agricultural householders, the gender of the household, the educational background of the household, the size of the household, types of farming practiced, involvement in the extension package program, feeding locations, agro-ecological zones, cattle healthcare practices, the land area owned by the household, vaccinations administered to cattle, and the region.

### Methods of data analysis

In this study, multilevel analysis was conducted with the expectation that there would be a difference in the number of cattle deaths per household across the region. Multilevel models allow the relationship between the explanatory variables at different levels and dependent variables at lower levels to be estimated, enabling the extent of variation in the outcome of interest to be measured at each level assumed in the model both before and after the inclusion of the explanatory variables in the model [31]. Two hierarchies were stated (for instance households and regions) in a multilevel logistic regression model. Units at one level are nested within units at the next higher level The households are level-1 and the regions are level-2. Intra-class correlation coefficient (ICC) represents the proportion of the total variance that is used to test heterogeneity in between-groups and it provides an assessment of whether or not significant between-groups variation exists. There are different count regression models to analyze data with count-dependent variables. The Akaike information criterion (AIC) and Bayesian information criterion (BIC) are methods used to evaluate models and select an appropriate fitted model. A model with a lower AIC and BIC is preferred over a model with a larger AIC and BIC.

The response variable is a count variable and the variance of the response variable is greater than the mean of the response variable, we use a negative binomial model. Sometimes when analyzing a response variable that is a count variable, the number of zeroes may seem excessive A standard negative binomial model would not distinguish between these two processes, but a zero-inflated model allows for and accommodates this complication. When analyzing a dataset with an excessive number of outcome zeros and two possible ones that arrive at a zero outcome, a zero-inflated model should be considered. Zero-inflated negative binomial (ZINB) regression models handle data with overdispersion and excess zeros. However, with hierarchical study designs/nested data, overdispersion and lack of independence may occur [30].

#### Software used.

The statistical software used in this study included SPSS version 20 and R^ + ^. SPSS was employed for data extraction, while R^ +^ was used for the analysis.

## Results and discussion

### Descriptive results

Table 1 shows that (86.37%) of agriculturalholders did not face any cattle death through the survey period and (13.63%) of agricultural householders faced at least one of their cattle death. The large numbers of zero outcomes in the data set led to a right-skewed distribution.

**Table 1:** Frequency distribution of numbers of cattle deaths per household.

Cattle death	Observation	Percent
0	26,410	86.37
1	2,602	8.51
2	784	2.56
3	314	1.03
4	159	0.52
5	110	0.36
6	52	0.17
7	43	0.14
8	23	0.08
9	7	0.02
10 +	72	0.24
Total	30,576	100.00

### Multilevel analysis

We investigate two-tiered count regression models in which households (level-1 units) are organized within 10 regions (level-2 units) to identify the factors that affect cattle mortality predictors and to explore the variations in cattle deaths among these regions.

#### Test of heterogeneity.

The intra-class correlation (ICC) with *ρ* =  0.146 indicates that 14.6% of the variance of the number of cattle deaths is at the grouping level (Region).

#### Model selection criteria.

Table 2 shows the values of AIC, BIC, and deviance for model selection and fit criteria. A lower value of these information criteria suggests a better fit. The Values of AIC, BIC, and deviance for the multilevel ZINB regression model are small as compared to other multilevel count regression models and we conclude that the multilevel ZINB regression model best fits the data.

**Table 2:** Compression of multilevel count model based on AIC, BIC, and deviance.

Criteria	Multilevel model					
	Poisson	NB	ZIP	ZINB	HP	HNB
AIC	41345.89	32688.44	34122.29	**32058.57**	34122.29	32267.80
BIC	41562.41	32913.29	34547.02	**32491.63**	34547.02	32700.85
Deviance	41293.9	32634.4	34020.3	**31954.6**	36938.3	32163.8

#### Compression of the model in multilevel ZINB regression.

The AIC, BIC, and deviance values are used to select the best fitting models among the three fitted multilevel ZINB regression models empty model, random intercept model, and random coefficients model.

In Table 3, the compared values of AIC, BIC, and deviance for the model with only random intercepts were 32002.15, 32443.54, and 31896.2, respectively. Since these AIC, BIC, and deviance values are lower than those of both the empty model and the random coefficients model, the random intercept model is more favorable than the random coefficients model.

**Table 3:** Compression of the model in multilevel ZINB regression.

Criteria	Multilevel model		
	Intercept-only model	Random-Intercept model	Random-coefficient model
AIC	34454.21	**32002.15**	32014.54
BIC	34495.85	**32443.54**	32489.23
Deviance	34444.2	**31896.2**	31900.5

#### Result of random-intercept ZINB count part.

The results in Table 4 showed, that Livestock farming types are statistically significant with cattle death per household, and the expected numbers of cattle deaths increase by a factor of $e^{\beta }$ = 2.314 when compared to croup farming types keeping other variables constant in the model. Untreated cattle show a statistically significant correlation with the number of cattle deaths per household, indicating that the rate of cattle mortality rises by 1.384 compared to treated cattle, assuming other factors remain constant in the model. Cattle that are not vaccinated are statistically significant with cattle death per household expected numbers of cattle deaths increase by a factor of 1.123 when compared to Cattle that are vaccinated considering other variables are constant in the model. Feeding area of cattle, such as grazing on holding, common pasture grazing, and others have a significant effect on cattle deaths per household that the expected numbers of cattle deaths increase by a factor of $e^{\beta }$ which is 1.411, 2.307, and 1.502 respectively when compared to in house/barn considering all other variable constants in the model. The level of informal education among households shows a statistically significant relationship with the number of cattle deaths per household, indicating that the anticipated cattle deaths rise by a factor of 1.167 when comparing households with no education, assuming other variables in the model remain constant. The land size of households 0.10-0.5 hectares and 5.01-10.0 hectares are statistically significant with the cattle death per household expected number of cattle deaths decreasing by 0.829 and 0.651 respectively when compared to households who have less than 0.1 hectare keeping other variables constant in the model. The estimated coefficient of household size is positive and had a significant effect on cattle death per household. That means the expected number of cattle deaths increases by a factor of 1.048 for every one-unit increase in the household size, holding another variable constant in the model. For every one unit increased household age the odds of cattle death increase by a factor of 1.004, considering all other variables constant in the model.

**Table 4:** Random–Intercept ZINB regression model count part.

Fixed effect for count part								
Variables	Categories	Estimate	Std. Error	z value	Pr(>|z|)	IRR	95% CI for *β*	
							lower	upper
	(Intercept)	−1.224	0.282	−4.335	0.000	0.294	−1.777	−0.670
Sex	Male (Ref)							
	Female	−0.045	0.063	−0.707	0.480*	0.956	−0.169	0.079
Farming Types	Ref(crops)							
	Livestock	0.839	0.191	4.400	0.000	2.314	0.465	1.213
	Both	−0.058	0.171	−0.342	0.732*	0.944	−0.393	0.276
Feeding area	House/barn (Ref)							
	Grazing on holding	0.344	0.073	4.710	0.000	1.411	0.201	0.487
	Common grazing	0.836	0.078	10.68	0.000	2.307	0.682	0.989
	Others	0.407	0.090	4.501	0.000	1.502	0.230	0.584
Treatment	Yes(Ref)							
	No	0.284	0.068	−4.182	0.000	1.328	−0.418	−0.151
Vaccination	Yes (Ref)							
	No	0.116	0.048	2.396	0.017	1.123	0.021	0.211
Agro-ecology zone	High hot (Ref)							
	Hot	−0.072	0.100	−0.716	0.474*	0.931	−0.268	0.124
	Low land	0.022	0.102	0.214	0.831*	1.022	−0.178	0.221
	Upland	−0.112	0.110	−1.013	0.311*	0.894	−0.327	0.104
	Cool	−0.841	0.591	−1.424	0.154*	0.431	−1.999	0.316
	High Cool	0.142	0.341	0.415	0.678*	1.153	−0.527	0.811
Education	No education (Ref)							
	Informal education	0.155	0.079	1.966	0.049	1.68	0.000	0.309
	Primary education	−0.013	0.056	−0.240	0.810*	0.987	−0.124	0.097
	Secondary & above	−0.133	0.075	−1.762	0.078*	0.875	−0.281	0.015
Household Land size	<0.1 hectare(Ref)							
	0.10–0.5 hectare	−0.187	0.091	−2.065	0.039	0.829	−0.365	−0.010
	0.51–1.00 hectare	−0.126	0.091	−1.386	0.166*	0.885	−0.304	0.052
	1.01–2.00 hectare	−0.145	0.089	−1.636	0.102*	0.865	−0.319	0.029
	2.01–5.00 hectare	−0.159	0.095	−1.678	0.093*	0.853	−0.344	0.027
	5.01–10.0 hectare	−0.429	0.174	−2.466	0.014	0.651	−0.771	−0.088
	over 10 hectare	−0.133	0.182	−0.733	0.464*	1.142	−0.490	0.223
Age	Age	0.004	0.002	2.478	0.013	1.004	0.001	0.007
HH- size	hh- size	0.047	0.009	5.020	0.000	1.048	0.029	0.065

#### Interpretation of random-intercept ZINB model for covariates of zero counts.

The covariates zero counts of the ZINB model indicate that Farming types, feeding areas, vaccination, agroecology zone, education, and Household size have a significant effect on the probability of being in the always zero group. From Table 5 the odds of being in zero group output cattle death for only livestock farming type and mixed farming types decreased by 0.332 and 0.134 respectively compared to crop farming types considering other variables constant in the model. The odds of being in zero group cattle death for other feeding areas increased by the factors of 2.48 compared to the feeding area of the barn considering other variables constant in the model. The likelihood of experiencing zero cattle deaths in the unvaccinated group increased by a factor of 1.736 compared to the vaccinated group, holding other variables in the model constant. The odds of being in zero group cattle death agro-ecology zone; Hot, Low land and High land decreased by the factors of 0.547, 0.666, and 0.313 respectively compared to High Hot considering other variables constant in the model. The odds of being in zero group cattle death for primary and secondary educational level increased by the factors of 1.32 and 1.456 respectively as compared to having no education level considering other variables constant in the model. The odds of being in zero group cattle death for one unit increase household’s age decreased by 0.993 considering other variables constant in the model. The odds of being in zero group cattle death for one unit increase household size decreased by the factors of 0.899 considering other variables constant in the model.

**Table 5:** Random–-Intercept ZINB regression model zero count part.

Zero-inflated part								
Variables	Categories	Estimate	Std. Error	z value	Pr(>|z|)	IRR	95% CI for *β*	
							Lower	Upper
	(Intercept)	−9.968	668.45	−0.015	0.988 *	0.000	−1320.1	1300.2
Sex	Ref(male)							
	(Sex)2	0.165	0.112	1.477	0.140 *	1.180	-0.054	0.385
Farming Types	Ref(crops)							
	Livestock	−1.125	0.237	−4.750	0.000	0.325	−1.589	−0.661
	Both	−2.078	0.214	−9.696	0.000	0.125	−2.498	−1.658
Feeding area	Ref(House/barn)							
	Grazing on holding	0.306	0.168	1.819	0.069 *	1.358	−0.024	0.636
	Common pasture grazing	−0.143	0.174	−0.820	0.412 *	0.867	−0.484	0.198
	Others	0.912	0.179	5.093	0.000	2.489	0.561	1.262
Treatment	Ref(yes)							
	No	13.058	668.436	0.020	0.984 *	468832	−1297.1	1323.2
Vaccination	Ref(yes)							
	No	0.552	0.127	4.349	0.000	1.737	0.303	0.800
Agro-ecology zone	Ref(High Hot)							
	Hot	−0.603	0.174	−3.473	0.001	0.547	−0.943	−0.263
	Low land	−0.406	0.179	−2.270	0.023	0.666	−0.756	−0.055
	High land	−1.163	0.196	−5.921	0.000	0.313	−1.549	−0.778
	Cool	−0.330	1.203	−0.274	0.784 *	0.719	−2.687	2.028
	High Cool	−2.743	1.799	−1.525	0.127 *	0.064	−6.268	0.782
Education	Ref(No education)							
	Informal education	0.329	0.169	1.953	0.051 *	1.390	−0.001	0.659
	Primary education	0.278	0.117	2.368	0.018	1.320	0.048	0.508
	Secondary and above education	0.376	0.152	2.474	0.013	1.456	0.078	0.673
Household land size	Ref(<0.1 hectare)							
	0.10–0.5 hectare	−0.077	0.185	−0.418	0.676 *	0.926	−0.440	0.285
	0.51–1.00 hectare	0.027	0.184	0.147	0.883 *	1.027	−0.334	0.389
	1.01–2.00 hectare	−0.236	0.185	−1.274	0.203 *	0.790	−0.600	0.127
	2.01–5.00 hectare	0.035	0.195	0.178	0.859 *	1.037	−0.348	0.418
	5.01–10.0 hectare	−0.321	0.378	−0.851	0.395 *	0.725	−1.061	0.419
	over 10 hectare	0.032	0.371	0.087	0.931 *	1.033	−0.694	0.759
Age	Age	−0.007	0.003	−2.377	0.017	0.993	−0.013	−0.001
HH_size	hh_size	−0.107	0.019	−5.505	0.000	0.899	−0.145	−0.069

## Discussion

In Ethiopia, agricultural activities are divided into three farming activities; crops, livestock, and mixed (both) farming [32]. Based on the ZINB regression model count part Table 4 shows that livestock farming types with cattle deaths per household increase 2.3 times compared to crop farming types. This result is consistent with [8,18], but contradict with [18]. The age of agricultural holders is significant for determinates of cattle death in Ethiopia with each yearly increase in the ages of agricultural holders the number of cattle deaths is increased by 4%. This result is contradicted by [32]. The estimated coefficient of household size is positive and had a significant effect on cattle death per household. This means that the number of livestock deaths increased by 4.8% for each increase in household size. This result is consistent with [18] and contradicted with [32]. Cattle that are not vaccinated are statistically significant with cattle death per household the expected number of cattle deaths increases by a factor of 12.3% when compared to cattle that are vaccinated. This result is consistent with [25]. Feeding areas (places) of cattle, such as grazing on holding, common pasture grazing, and others have a significant effect on cattle death per household that the cattle death increases when compared to cattle feeding in a house/barn. The land size of households 0.10-0.5 hectares and 5.01-10.0 hectares are statistically significant with the cattle death per household expected number of cattle deaths decreasing by 82.9% and 65.1% respectively when compared to agricultural households that have less than 0.1 hectare. This result is consistent with [23]. Untreated cattle show a statistically significant correlation with the number of cattle deaths per household, indicating that cattle mortality rises by 1.384 compared to treated cattle. This result is consistent with [33,34].

According to the result of the ZINB regression model, zero count part; Livestock, and mixed farming types have a significant effect on zero counts to decrease cattle death compared to crop farming types, and other feeding areas of cattle compared to house/barn to increase cattle death, agro-ecology zone such as Hot. Low land and High Land have effects compared to High Hot to decrease cattle death, Primary and secondary education levels of households have fator on zero count cattle death when compared to no education level of households, the number of cattle deaths decreased by a factor of 10.1% when an increase in household size, one unit of the age of agricultural households increase the number of cattle deaths is also decreased by 7%.

## Conclusions

A multi-level analysis was implemented with the expectation of the number of cattle deaths across the region. Before analyzing data using a multi -level approach, the difference in livestock death in different areas was evaluated using the class correlation (ICC). This indicated that 14.6% of the deaths of cows occur at the regional level. This suggests that there is variability in cattle deaths among the regions. Compared to single-level count regression models, the multilevel count regression model fits data better.

Comparing those models based on model comparison methods(criteria) among six multilevel count regression models; the multilevel ZINB regression model was the most appropriate model to fit the cattle death per household.

In the positive count part of the random-intercept ZINB regression model, the variables like; farming types, cattle feeding area, treatment, vaccination, householder land size, age of household, household size, and education level had statistically significant effects on cattle death. Based on this study’s findings, the Ministry of Agriculture and relevant stakeholders should take the identified causes of cattle mortality into account when formulating a policy aimed at decreasing cattle deaths in Ethiopia. The cattle death per household significantly varies from region to region. Thus it might be good that future studies focus on identifying the risk factors of cattle death in each region of Ethiopia. The Ministry of Agriculture should take appropriate initiatives to create awareness among farmers regarding cattle vaccination and improve veterinary services in the country. Promote a mixed farming system rather than only a livestock farming system to decrease cattle death.

## Supporting information

S1 Data Data file. (ZIP)

## Abbreviations

AIC: Akaike Information Criteria
BIC: Bayesian Information Criteria
CSA: Central Statistical Agency
HNB: Hurdle – Negative Binomial
HP: Hurdle –Poisson
ICC: Intra-class Correlation Coefficient
NB: Negative Binomial
ZIP: Zero-Inflated Poisson
ZINB: Zero-Inflated Negative Binomial
